# Andragogic Model Curriculum for One-Year ACGME-Accredited Fellowship Programs: Single-Center Educational Improvement Project

**DOI:** 10.2196/81570

**Published:** 2026-06-23

**Authors:** Romy Yun, Allyson Farran, Echo Rowe, Dina Sheira, Christian Jackson, Thomas J Caruso

**Affiliations:** 1 Department of Anesthesiology, Perioperative, and Pain Medicine, Stanford University School of Medicine, Center for Academic Medicine, 453 Quarry Road, MC 5663, Palo Alto, CA, 94304, United States, 1 (650) 723-6415; 2 Department of Anesthesiology, Perioperative, and Pain Medicine, University of Alberta School of Medicine, Edmonton, AB, Canada; 3 Stanford University School of Medicine, Stanford, CA, United States

**Keywords:** education, medical, curriculum, learning, fellowship

## Abstract

**Background:**

The number of 1-year Accreditation Council for Graduate Medical Education (ACGME) fellowships continues to grow. The ACGME recommends a holistic curriculum with nonclinical areas, inclusive of educational sessions. Given the competing demands between clinical skill development, educational pursuits, and work-hour restrictions, we propose an andragogic curriculum using pediatric anesthesiology as the model fellowship.

**Objective:**

The primary objective was to improve fellows’ perceptions of their educational experience during their fellowship year after implementing an andragogic holistic curriculum. Secondary objectives assessed improvements in diversity, equity, and inclusion (DEI) training and resources.

**Methods:**

This was a single-center educational improvement project completed at Lucile Packard Children’s Hospital Stanford. Data were collected between 2014 and 2024. The new curriculum was introduced in 2021‐2022 and involved 12 different teaching modalities rooted in andragogic principles. A statistical process control p-chart was used to analyze the primary outcome based on the ACGME annual program evaluation. Outcomes were analyzed using censored regression modeling or a *t* test, depending on the presence of ceiling effects.

**Results:**

From 2014 to 2024, 58 of 60 pediatric anesthesiology fellows completed the ACGME survey. A break in the statistical process control p-chart for educational content scores occurred during 2021‐2022, when the new curriculum was introduced. The mean difference was 0.89 (*P*<.001). Scores in DEI improved (mean difference 0.52; *P*=.03), and no difference was noted in resources (mean difference −0.13; *P*=.98).

**Conclusions:**

Introduction of an andragogic curriculum into a pediatric anesthesiology fellowship program was associated with more favorable perceptions of educational content and DEI training.

## Introduction

Fellowship programs have expanded to meet the demand for subspecialized physicians [1]. One-year Accreditation Council for Graduate Medical Education (ACGME) programs continue to increase, with 75 currently registered [2]. Historically, fellowship training has been taught through clinical apprenticeships and didactics, which are resource and time intensive [3,4]. While subspecialty programs yield clinical competency, expertise in nonclinical areas has been less emphasized.

The ACGME defines core competencies and specialty-specific milestones for fellowship programs. Expanded ACGME milestones include quality improvement; diversity, equity, and inclusion (DEI) training; well-being; and dedicated resources for scholarly activity. Despite short durations of fellowships compared to residencies, ACGME programmatic requirements are relatively similar, creating challenges for program directors seeking to meaningfully incorporate these requirements [5,6]. Given the increasing number of ACGME milestones, an andragogic approach may provide an effective framework and incorporate individual learning styles across trainees [3]. This approach leverages the previous experiences of a learner and puts them in a more active role within the education process. Using established curricular frameworks from Knowles’ Andragogic Model and Kolb’s Experiential Learning Cycle, we developed an enhanced fellowship curriculum that leverages andragogic principles to integrate ACGME clinical and nonclinical curricular recommendations into a single year. This approach tailors the experience to mature learners with an active role in their educational experience [3,7].

Although this study describes the integration of all ACGME competencies into a 1-year curriculum using the Pediatric Anesthesiology Fellowship as the model, the components are relatable to other ACGME programs. The primary aim of this curricular redesign was to improve fellows’ perceptions of their educational experience.

## Methods

### Setting

This was an educational improvement project completed between 2014 and 2024 at Stanford University School of Medicine within the ACGME-approved Pediatric Anesthesiology Fellowship. The fellows practice at Lucile Packard Children’s Hospital Stanford, a level-one trauma center with 20 operating rooms. The training occurs over a single year, with approximately 8 fellows in postgraduate year five or greater.

### Intervention

During the 2021‐2022 year, an andragogic curriculum was integrated into the fellowship program. Before this new curriculum, fellow education consisted of after-hours didactics, one simulation session, and an apprenticeship model of clinical care. The new curricular framework relied on Knowles’ Andragogic Model combined with Kolb’s Experiential Learning Cycle [8,9]. These models were preferred for their established application in health professions education and direct alignment with the characteristics of the fellowship learner population. Fellows enter the program as fully qualified physicians with professional identities, diverse prior clinical experiences, and intrinsic motivation to achieve subspeciality competence. Knowles’ Andragogic Model was chosen for its emphasis on the role of the independent self-directed learner, prioritized problem-centered approaches, and application of learning into daily life [8]. Kolb’s Experiential Learning Cycle was selected as a complementary framework since it provides a mechanistic account of how learning occurs within the experiential environment [9,10]. While Knowles’ principles guided the overall curriculum design and ensured that it was self-directed, problem-centered, and responsive to fellows’ prior experience, Kolb’s cycle was used to structure the learning process itself. Specifically, the curriculum was designed for concrete clinical experiences to be systematically followed by structured reflection, didactic conceptualization, and opportunities for active experimentation, rather than solely relying on experience.

Curricular components with various delivery platforms were implemented that prioritized the unique characteristics of fellowship-level learners and the structured process by which direct clinical experiences are converted into meaningful learning. A 12-component syllabus that addressed clinical and nonclinical ACGME requirements was developed, which included experiential, self-directed, problem-based, and asynchronous remote learning platforms (Table 1) [11]. Within these 12 learning modalities and trainings, each curricular component was curated around chapters in a foundational pediatric anesthesiology textbook and core knowledge requirements dictated by ACGME pediatric anesthesiology milestones [12]. Learning modalities within the new curriculum included directed readings, didactic lectures, quality improvement training, problem-based learning discussions, simulation, workshops, professional and behavioral health series, doctoring with care series, oral board preparation series, DEI training, journal clubs, and self-assessment in addition to daily clinical care responsibilities (Multimedia Appendix 1). Andragogic principles such as self-concept, need to know, problem-centered orientation, prior experience, readiness to learn, and internal motivation were applied to the learning modalities within the new curriculum (Table 1). The principle of problem-centered orientation was embedded through case-based conferences, simulations, and workshops. Internal motivation was applied to coaching conversations, and reflections focused on growth and ownership of a quality improvement training module and a quality improvement project of their choosing. Didactic sessions and directed readings highlighted the need to know and readiness to learn principles with explicit framing of clinical context. Additionally, the stages within Kolb’s Experiential Learning Cycle (concrete experience, reflective observation, abstract conceptualization, and active experimentation) were used within the curricular components to ensure that clinical experiences were followed by thoughtful reflection, feedback, and conceptualization (Table 1).

**Table 1.** Integration of andragogic curricular components.

Knowles’ principles	Kolb’s stages	Curricular component	Description	Level of integration
Need to know and readiness to learn	Abstract conceptualization	Directed readings	Weekly topic readings correlated to organ-based learning blocks from core textbook	Asynchronous
Need to know and readiness to learn	Abstract conceptualization	Didactic lectures^a^	Approximately 25 midday lectures with topics aligned with readings/questions in organ-based blocks	In-person or remote
Internal motivation	Abstract conceptualization	QI^b^ training	QI web-based modules Morbidity and mortality monthly conferences	Asynchronous and in-person or remote
Problem-centered orientation and prior experience	Abstract conceptualization	Problem-based learning discussions^a^	Case-based discussions correlated to organ-based learning blocks	In-person or remote
Problem-centered orientation and prior experience	Abstract conceptualization and active experimentation	Simulation^a^	Case-based scenarios practicing technical and nontechnical skills In situ, virtual, and extended reality simulations offered	In-person and experiential
Problem-centered orientation and readiness to learn	Abstract conceptualization	Workshops	Hands-on educational workshops Examples: pacemaker, point of care ultrasound, regional anesthesia cadaver lab, vascular access	In-person
Internal motivation	Reflective observation	PBHS^c^ and DWCS^a^^,^^d^	DWCS: Opportunities for reflection, self-compassion, community-building PBHS: Roundtable discussions on physician well-being, finances, nutrition, sleep deprivation, burnout	In-person
Self-concept and internal motivation	Abstract conceptualization and reflective observation	Oral board preparation series	Interactive sessions geared toward oral board preparation Led by current faculty oral board examiners	In-person and interactive learning
Internal motivation and need to know	Reflective observation	DEI^e^ training^a^	DEI course sequence occurring every other month Topic examples: health disparities, use of perioperative interpreters, gender diverse patients, racial disparities in medicine	In-person and introspective learning
Prior experience and need to know	Abstract conceptualization and reflective observation	Journal clubs^a^	Group-based discussions led by trainees on selected journal articles	In-person or remote and flipped classroom
Self-concept	Reflective observation	Self-assessment	Assigned practice questions aligned with readings in organ-based learning blocks Mid and end of year self-assessment exams similar to the pediatric anesthesiology board exam questions Board specific in-training exam	Asynchronous and in-person or remote

a During workday hours.

b QI: quality improvement.

c PBHS: professional and behavioral health series.

d DWCS: doctoring with care series.

e DEI: diversity, equity, and inclusion.

### Outcomes and Data Collection

The primary outcome assessed fellows’ perception of educational content. This was selected because it demonstrated a broad representation of nonclinical components highlighted by ACGME guidelines. Secondary outcomes explored DEI training and programmatic resources. The DEI category was selected since it assessed reflective practice and inclusivity, and the resources category assessed the program’s educational infrastructure.

Outcomes were measured with the ACGME trainee survey, which assesses education quality and compliance with ACGME requirements [13]. Sections within the ACGME survey are standardized into preset categories. The educational content subsections include a broad range of questions on well-being, burnout, and scholarly activities. The resources subsections assess protected time for educational and professional activities and overall institutional support. The DEI subsections include questions regarding inclusivity, diversity of patient populations, and engagement of a program’s DEI efforts. Survey responses are based on a 5-point Likert scale and are annually completed by the fellows. ACGME fellow survey results from 2014 to 2024 were collected from the educational content and resources categories on the survey. Data were collected from 2019 to 2024 regarding DEI training, a new category introduced in 2019. Each category contained several subsection scores, which were averaged by the ACGME to provide the overall category mean.

### Statistical Analysis

To analyze the primary outcome, a statistical process control p-chart monitored a change in fellows’ responses to the educational content subsection of the ACGME survey. The Western Electric rules assessed special cause variation [14]. Rule one in Western Electric states that special cause variation occurs when a data point falls outside the 3 SD upper control limit from the center line. This occurred in the 2021‐2022 academic year, leading to the establishment of a new center line. Mean outcomes were assessed using censored regression modeling if there was a ceiling effect or a *t* test if no ceiling effect. A *P* value less than .05 was considered significant. All analyses were performed using Excel (Microsoft Corporation) and R statistical software (version 4.5.1; R Foundation for Statistical Computing; *censReg* package) [15].

### Ethical Considerations

This project received a waiver of consent by the Stanford University Institutional Review Board (eProtocol No. 87506). Data were deidentified by the ACGME before reporting the results to the program. There was no compensation given to participants.

## Results

### Demographics

Of the 60 pediatric anesthesiology fellows, 58 completed the ACGME survey from 2014 to 2024, with an overall response rate of 97%. During the data collection period, 56% (n=34) of the fellows identified as female and 44% (n=26) as male.

### Primary Outcome

Special cause variation was noted in the statistical process control p-chart for mean scores for educational content during the 2021‐2022 academic year, which coincides with the implementation of the new curriculum. Given a ceiling effect, censored regression was used to estimate the mean difference. A mean difference of 0.89 (SE 0.27) from 2014 to 2021 was observed compared to 2022‐2024 (*P*<.001).

### Secondary Outcomes

The mean difference between DEI training scores did not exhibit a ceiling effect; therefore, a *t* test was used to analyze the results. For DEI training (n=36), we demonstrated a difference of 0.52 (SE 0.2; *P*=.03). Fellows’ perceptions of program resources (n=58) had a ceiling effect, and no estimated difference was observed (mean difference −0.13, SE 0.24; *P*=.98).

## Discussion

Holistic integration of all ACGME curricular sections is challenging to implement in shorter-duration training programs. This project assessed learners’ perception of an enhanced pediatric anesthesiology fellowship curriculum. We relied on multiple andragogic principles on established curricular frameworks to guide the integration of recommended ACGME components. The curriculum improved fellows’ perception of their educational experience and their DEI training, with no difference in perception of programmatic resources.

The key components of the enhanced curriculum expanded on didactics to include several curricular threads grounded in andragogic learning theories. Self-directed, asynchronous learning improves trainee engagement and provides a sense of agency [8,16,17]. We used a set of professionally created online modules to teach quality improvement methodology, which fellows completed at their own pace. Additionally, self-directed readings with affiliated questions were aligned with organ-based learning blocks throughout the year. Experiential learning provided opportunities to build knowledge and skill sets in realistic clinical scenarios with structured feedback via case-based problem-based learning discussions, procedural skills workshops, and simulations [18]. For our simulation sessions, the fellows experienced in situ simulations that occurred during typical working hours in their practice environment. Because integration of curriculum components into the workday is associated with improved trainee perception of work-life balance, several curricular components were held during daytime hours [19,20]. Additionally, a professionalism and behavioral health monthly thread was developed to improve resiliency and self-care [21].

Implementation of DEI curricula remains challenging with limited evidence on best practices to guide effective strategies to fulfill ACGME common program requirements [22]. In this report, the improvement in DEI training was due to two new core curricular components. These components were influenced by special study modules, which are programs that intentionally provide educational opportunities to explore topics beyond the core curriculum [23]. Understanding that adult learners value critical thinking, introspection, and clinical application, the DEI sessions consisted of monthly midday lectures that challenged fellows’ implicit biases and explored social determinants of health within diverse patient populations. By also including dedicated time for self-reflection, called doctoring with care, fellows were provided the opportunity to process challenging patient and personal interactions [21]. Implementation of well-designed modules alongside interactive learning environments increased fellow satisfaction within these domains. However, the enhanced curriculum did not demonstrate improvements in programmatic resources. Although surprising, variation within subsections in this category may account for the lack of difference.

This project had several limitations. First, inherent to most improvement projects, there was no control group. However, the demographic constituency of the fellows did not change during the project period. Second, while standardized across the country, there is annual variation in the ACGME subsection questions. Despite this variance, the ACGME routinely uses its survey to highlight programmatic trends, similar to our methodology. Third, this enhanced curriculum was applied to a single fellowship. Although we believe this specific andragogic approach would be beneficial for other shorter-duration programs, this hypothesis is untested. Fourth, there was no method to account for natural shifts that occur over time, such as ACGME expectations, institutional culture, or national attention to DEI, that could explain these improvements. Fifth, there is a wide range of other unmeasured items that could impact trainee experience not captured by the ACGME survey. Lastly, our data relied on self-reported perception scores from the ACGME survey. Although we intentionally chose a standardized measure to improve the generalizability of our findings to other national training programs, we did not include objective educational outcomes such as examination scores. Future studies should investigate if these changes translate into measurable improvements in fellow performance and whether these gains are practically meaningful.

Introduction of an andragogic curriculum to the Pediatric Anesthesiology fellowship program was associated with more favorable perceptions of the educational experience. Adoption of aspects of this curricular framework may improve the educational content of other shorter-duration training programs. Future studies will explore additional curricular enhancements and their effects on 1-year training programs beyond pediatric anesthesiology to better control for potential confounders and natural variations within ACGME expectations.

## Supplementary material

10.2196/81570 Multimedia Appendix 1 Learning modalities within the new curriculum.

## Abbreviations

ACGME: Accreditation Council for Graduate Medical Education
DEI: diversity, equity, and inclusion
