# Supplementary figures and images for: Andragogic Model Curriculum for One-Year ACGME-Accredited Fellowship Programs: Single-Center Educational Improvement Project
Source: JMIR Med Educ. 2026 Jun 23;12:e81570. doi: 10.2196/81570 (PMC13290103; doi:10.2196/81570)

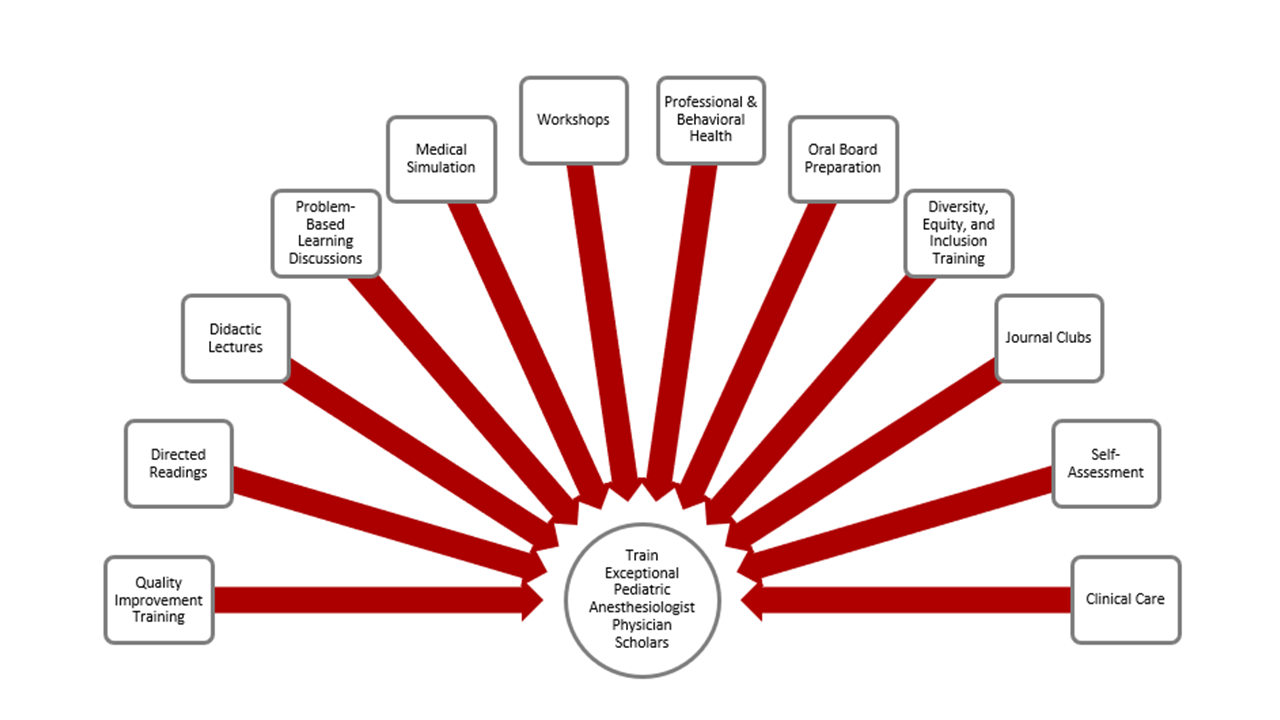

Supplement: Multimedia Appendix 1 [file mededu-v12-e81570-s001.png]
